# Motor Cortex Theta and Gamma Architecture in Young Adult APPswePS1dE9 Alzheimer Mice

**DOI:** 10.1371/journal.pone.0169654

**Published:** 2017-01-10

**Authors:** Anna Papazoglou, Julien Soos, Andreas Lundt, Carola Wormuth, Varun Raj Ginde, Ralf Müller, Christina Henseler, Karl Broich, Kan Xie, Britta Haenisch, Dan Ehninger, Marco Weiergräber

**Affiliations:** 1 Department of Neuropsychopharmacology, Federal Institute for Drugs and Medical Devices (Bundesinstitut für Arzneimittel und Medizinprodukte, BfArM), Bonn, Germany; 2 German Center for Neurodegenerative Diseases (Deutsches Zentrum für Neurodegenerative Erkrankungen, DZNE), Bonn, Germany; 3 Department of Psychiatry and Psychotherapy, University of Cologne, Cologne, Germany; Nathan S Kline Institute, UNITED STATES

## Abstract

Alzheimer’s disease (AD) is a multifactorial disorder leading to progressive memory loss and eventually death. In this study, an APPswePS1dE9 AD mouse model has been analyzed for motor cortex theta, beta and gamma frequency alterations using computerized 3D stereotaxic electrode positioning and implantable video-EEG radiotelemetry to perform long-term M1 recordings from both genders considering age, circadian rhythm and activity status of experimental animals. We previously demonstrated that APPswePS1dE9 mice exibit complex alterations in hippocampal frequency power and another recent investigation reported a global increase of alpha, beta and gamma power in APPswePS1dE9 in females of 16–17 weeks of age. In this cortical study in APPswePS1dE9 mice we did not observe any changes in theta, beta and particularly gamma power in both genders at the age of 14, 15, 18 and 19 weeks. Importantly, no activity dependence of theta, beta and gamma activity could be detected. These findings clearly point to the fact that EEG activity, particularly gamma power exhibits developmental changes and spatial distinctiveness in the APPswePS1dE9 mouse model of Alzheimer’s disease.

## Introduction

Alzheimer’s disease (AD) is a multifactorial neurodegenerative disorder resulting in progressive cognitive decline and memory loss. Histologically, AD is characterized by extracellular amyloid plaques based on the excessive accumulation of amyloid beta (Aβ) peptides in the central nervous system (CNS) [1–3]. Aβ peptides are cleavage products derived from the amyloid precursor protein (APP) via sequential endoproteolysis by specific secretases, i.e. beta-site amyloid precursor protein cleaving enzyme 1 (BACE-1) and γ-secretase [4]. The length of Aβ peptides ranges from 36–43 amino acids [5]. In general, the abundance of Aβ_1–40_ is higher compared to Aβ_1–42_, the latter being prone to aggregate and exhibiting enhanced cytotoxic effects [6]. Various APP mutations, such as Swedish double mutation KM670/671NL were reported to be pro-amyloidogenic as they can facilitate the generation of toxic Aβ_1–42_ peptides [7]. In addition, mutations in presenilin (PS)-1 and 2 that serve as catalytic sites for γ-secretase, can further aggravate the production of pro-amyloidogenic Aβ_1–42_ [8]. Apart from the Aβ plaque formation, AD neurons can also exhibit neurofibrillary tangles resulting from intraneuronal deposits of hyperphosphorylated tau (τ) protein [9, 10].

Numerous transgenic mouse models of AD supposed to fulfill the criteria of homology, isomorphism and predictability have been generated in the past [11, 12]. These models display age-related AD-specific alterations such as Aβ plaques, axonal and synaptic dystrophy, reduced synaptic plasticity and impaired learning and memory function [13–15]. Here we use an APPswePS1dE AD mouse model which is characterized by the Swedish double mutation (APPswe) cointegrated with human PS1 with exon 9 deletion (PS1dE9) [16–18]. These mutations result in overproduction of APP and PS1 splice variants with subsequent increase in neural Aβ load. Furthermore, transgenic mice display Aβ_1–42_ overload which might be associated with increased mortality and sudden death [19–21]. Based on the proictogenic effect of intracellular Aβ accumulation, it has been speculated that seizure activity might be responsible for sudden death in this model [22, 23]. APPswePS1dE9 mice develop first Aβ plaques around 4 month of age, particularly in the cortex and hippocampus. This coincides with a mortality peak around 3–4 months of age [24, 25]. At the age of 6 months memory deficits in radial arm water maze are prominent [26] whereas at 12 months, mice start exhibiting behavioral and cognitive deficits detectable in spatial navigation, reference learning and Morris water maze.

Cognitive alteration and learning and memory deficits are accompanied by complex central dysrhythmia, particular within the cortex and septohippocampal system [27] affecting theta and gamma activity [27]. Previous studies have investigated the electrical activity and specific frequency characteristics from electrocorticograms and other deflections in APP transgenic mice [4, 21, 22, 28–30]. Recent investigations [21, 30] focused on the analysis of early Alzheimer’s disease stages (animals aged 2.5–4.5 months) as this critical time range marks the first appearance of amyloid plaques. Lately, we performed a long-term radiotelemetric study of hippocampal frequency characteristics in young adult (14–19 wks old) APPswePS1dE9 mice using a Fast Fourier Transformation (FFT) based approach [31]. Automatic seizure detection unraveled severe gender-specific electroencephalographic seizure activity in both M1 and CA1 deflection. Seizure activity in APPswePS1dE9 exhibited high variability as has been reported for other AD mouse models before. Importantly, hippocampal EEG frequency analysis elicited complex age, gender and activity dependent alterations in the theta and gamma range [31]. Females displayed an antithetic decrease in theta (θ) and increase in gamma (γ) power at 18–19 weeks of age whereas related changes in males appeared earlier at 14 weeks of age. Furthermore, θ and γ power alterations in female APPswePS1dE9 turned out to be most prominent in the inactive state suggesting an impairment of atropine-sensitive type II theta in APPswePS1dE9 mice. These results clearly demonstrate that systemic electrophysiological alterations occur before any clinical signs of Alzheimer’s disease can be detected in these mice.

Here we present a systematic FFT-based frequency analysis and multi-parameter, i.e. gender, age and activity dependent longitudinal investigation of θ, β and γ activity in the cortical M1 EEG under unrestrained long-term recording conditions in young adult (14–19 wks old) APPswePS1dE9 mice.

## Materials and Methods

### Study animals

In this study APPswePS1dE9 transgenic mice with a C57BL/6J background carrying a human APP with Swedish double mutation (APPswe) cointegrated with human PS1 with exon 9 deletion (PS1dE) were used [16, 17]. The experimental animals (B6.Cg-Tg(APPswe, PSEN1dE9)85Dbo/Mmjax, MMRRC stock no. 34832-JAX) were purchased from Jackson Laboratory. In total, 21 control animals (10 ♂, body weight: 26.94 ± 0.64 g; 11 ♀, body weight: 21.23 ± 0.53 g) and 20 APPswePS1dE mice (9 ♂, body weight: 26.16 ± 0.56 g; 11 ♀, body weight: 21.73 ± 0.35 g) were analyzed in this study gender-specific. All experimental animals were housed in groups of 3–4 in clear Makrolon cages type II with ad libitum access to drinking water and standard food pellets. Mice were maintained at a temperature of 21 ± 2°C, 50–60% relative humidity, and on a conventional 12h light/dark cycle beginning at 5:00 a.m. using ventilated cabinets. All animal experimentation was performed in accordance with the National Institute of Health Guide for the Care and Use of Laboratory Animals (NIH Publications No. 80–23) revised 1996 or the UK Animals (Scientific Procedures) Act 1986 and associated guidelines, or the European Communities Council Directive of 24 November 1986 (86/609/ EEC) and of September 22^nd^, 2010 (2010/63/EU). Experiments were carried out according to the Guidelines of the German Council on Animal Care and all protocols were approved by the Local Institutional and National Committee on Animal Care (Landesamt für Natur, Umwelt und Verbraucherschutz, LANUV, Germany). Special attention was paid to minimize the animal sample size and the suffering of mice.

### Radiofrequency transmitter implantation for EEG recording

Mice were anesthetized using the volatile narcotic isoflurane (Baxter 100% V/V). Isoflurane was applied via facemask using a Matrix TM VIP 3000 Calibrated Vaporizer and a scavenger system from Harvard apparatus (USA) [32–34]. The radiofrequency transmitter TL11M2-F20-EET (2-channel transmitter, Data Science International (DSI, Germany), specifications: weight 3.9 g, volume 1.9 cc, input voltage range ± 1.25 mV, amplification factor (voltage gain) 200; nominal sampling rate 250 Hz) was implanted into a subcutaneous pouch on the back of the experimental animals. The EEG electrodes of the radiotelemetry transmitter were stereotaxically positioned via a computerized 3D stereotaxic StereoDrive system (Neurostar, Germany) [32–34].

### Epidural electrode placement for electrocorticographic recordings

The differential epidural surface electrode of channel 1 of the TL11M2-F20-EET transmitter was positioned at the following stereotaxic coordinates referring to the bregma craniometric landmark: (+)-lead, cranial +1 mm, and lateral of bregma 1.5 mm (left hemisphere). The differential electrode targets the primary motor cortex (M1). An epidural reference electrode was placed on the cerebellar cortex at bregma -6 mm, lateral of bregma 1mm (left hemisphere). Channel 2 of the transmitter was used for deep, intracerebral EEG recording from the hippocampal CA1 region the results of which were reported elsewhere [27, 33, 34]. The electrodes were fixed using glass ionomer cement (Kent Dental, UK) and the scalp was closed using over-and-over sutures (Ethilon, 6–0). As mice are predisposed to hypothermia, supplemental warmth was given to the animal with a heating pad during the whole surgical procedure. A detailed description of the stereotaxic electrode placement and transmitter implantation were previously described in detail [32–34]. For postoperative pain management, Carprofen (5 mg/kg, Rimadyl, Parke-Davis/Pfizer, Germany) was administered subcutaneously. Mice were given 10 days post-surgery to fully recover. This recovery period is based on the observation that no differences in basic physiological / behavioral parameters such as food and water uptake, motor activity, body temperature etc. could be detected between radiotransmitter implanted, non-implanted, and sham-operated mice 10 days post surgery [35].

### Confirmation of EEG electrode placement

To verify whether electrodes were properly placed in the cortical M1 region, brains were extirpated post-mortem and fixed in 4% paraformaldehyde. Subsequently, brains were cut to 60 μm slices using a Vibroslice Tissue Cutter EMS 5000-MZ (Campden Instruments Limited, UK). Slices were hematoxylin-stained for potential damage or impingement of the cortex. Animals that exhibited cortical damage were excluded from analysis.

### Radiotelemetric EEG data acquisition

The first long-term recording of 48 hrs was performed at day 10 post-surgery from the primary motor cortex (M1). A second 48 hrs long-term recording was performed at day 17 post-implantation using both deflections. For EEG data acquisition, the Dataquest ART 4.2 software (DSI) was used. No a priori filter cutoffs were applied. The nominal sampling rate (f) of the TL11M2-F20-EET transmitter is 250 Hz. Analysis was performed up to 70 Hz, considering the transmitter specific bandwidth and the Nyquist-Shannon limit of 125 Hz for this transmitter type. Note that high quality EEG recordings can be obtained for up to 8 wks. This limitation is due to ossification processes from the burred drilled holes that can lift the electrodes and induce EMG or ECG contamination.

### Radiotelemetric activity recording and analysis

As the animal moves about in its cage, the telemetry signal transmitted to the receiver antennas varies in strength. The signal strength may vary due to orientation of the animal relative to the receiver, or due to the distance of the animal from the receiver antennas. When the signal strength changes by a certain amount, an activity count is generated. The number of counts generated is dependent on both distance and speed of movement (acceleration). Note that the activity data provided by Dataquest A.R.T. is a relative measure of locomotor activity. Activity analysis was carried out for controls and APPswePS1dE9 mice from both genders.

### Analysis of electrocorticographic data

Recordings (48 hrs) of spontaneous EEG activity were performed based on a nominal sampling rate of 250 Hz of the radiofrequency transmitter. EEG data were FFT analyzed using NeuroScore 2.1 (DSI) in the frequency range of 0.5–70 Hz, comprising the typical delta (0.5–4 Hz), theta (4–8 Hz), beta (12–30 Hz) and gamma bands of i) 30–50 Hz and ii) 50–70 Hz. The upper gamma limit (70 Hz) is still below the Nyquist-Shannon limit of 125 Hz, thus FFT based analysis is valid [36]. The length of the individual EEG epochs that were FFT analyzed was 2 s. Subsequently, mean relative EEG power [%] was calculated for the individual frequency ranges, for both genders and the individual circadian stages, i.e. two dark (D1, D2) and two light cycles (L1, L2). In addition, activity data of mice during the conventional 12h light/dark cycle (starting at 5:00 a.m.) were used to correlate activity in different EEG frequency bands from both deflections with either the active (activity units > 0) or inactive state (activity units = 0).

Data were statistically analyzed and displayed as mean ± SEM. Statistics for frequency analysis were carried through by multiple Student’s t-test, corrected for multiple comparison using the Holm-Sidak method. Most of the statistics and graph presentations were performed with GraphPad Prism 6 for Windows.

## Results

### Theta frequency analysis in controls and APPswePS1dE9 mice

**Fig 1:**
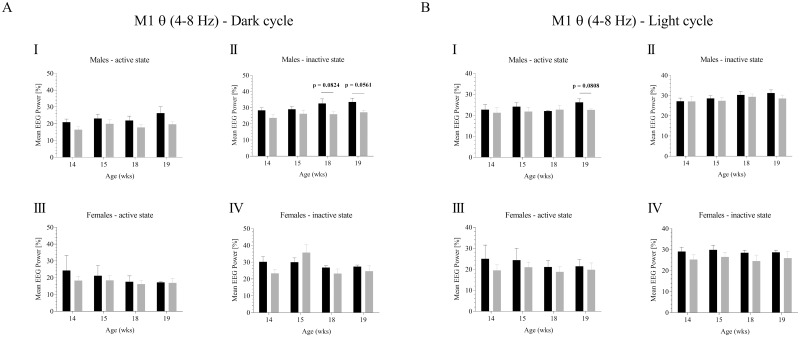
Theta frequency analysis of cortical M1 EEG recordings in controls and APPswePS1dE9 mice. The mean relative EEG theta power [%] was calculated FFT based for males and females considering potential circadian rhythmicity (dark phase (**A**), light phase (**B**)). Frequency analysis was performed for all four ages (14, 15, 18, 19 wks). Black, controls; gray, APPswePS1dE9. Note that only animals displaying highest quality EEGs (no EMG/ECG contamination) were finally included into the analysis. For sample size see original data.

Relative theta frequency power was analyzed in the range of 4–8 Hz for the light and dark cycle for 14, 15, 18 and 19 wks of age (Fig 1; S1 and S2 Files). We further analyzed the influence of the activity stage. Note that no differences in relative activity could be detected for any of the ages and circadian cycles studied in APPswePS1dE9 mice and controls [31]. Male APPswePS1dE9 mice exhibited significant increase in relative activity in the light, dark and total circadian rhythm at the age of 18 wks. No further alterations were detected [31]. Importantly, in contrast to the complex alterations in hippocampal theta which we reported previously, no significant alterations were detected in motor cortex (M1) theta activity in APPswePS1dE9. Statistical trends were observed in inactive males in the dark cycle at the age of 18 and 19 wks (32.600 ± 2.834 v. 25.975 ± 1.443, p = 0.0824; 33.515 ± 2.381 v. 27.138 ± 1.272, p = 0.0561; Fig 1A_II_) and in active males in the light cycle at the age of 19 wks (26.190 ± 1.621 v. 22.578 ± 0.583, p = 0.0808, Fig 1B_I_).

### Gamma frequency analysis in controls and APPswePS1dE9 mice

**Fig 2:**
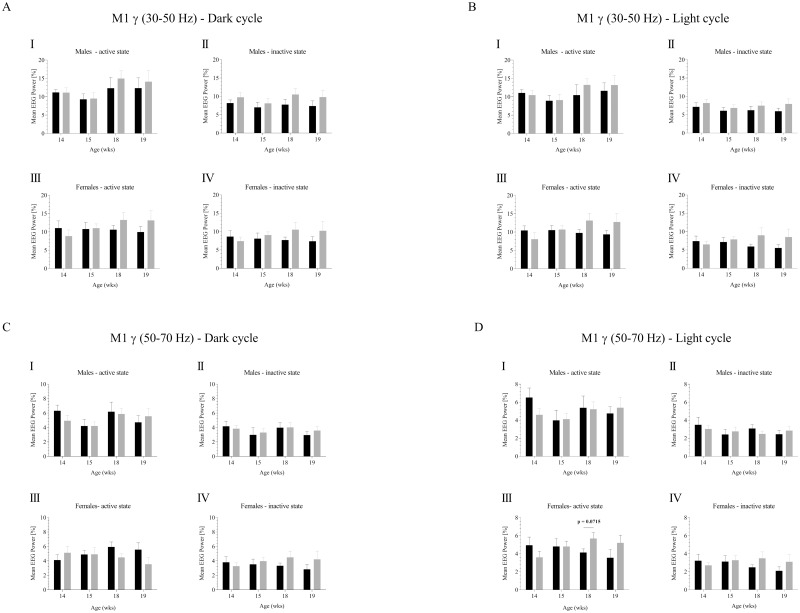
Gamma frequency analysis of cortical M1 EEG recordings in controls and APPswePS1dE9 mice. The mean relative gamma EEG power [%] was calculated FFT based for males and females considering potential circadian rhythmicity (dark phase 30–50 Hz (**A**), light phase 30–50 Hz (**B**), dark phase 50–70 Hz (**C**), light phase 50–70 Hz (**D**)). Frequency analysis was performed for all four ages (14, 15, 18, 19 wks). Black, controls; gray, APPswePS1dE9. Note that only animals displaying highest quality EEGs (no EMG/ECG contamination) were finally included into the analysis. For sample size see original data.

Relative gamma frequency power was analyzed in the range of 30–50 Hz for the dark (Fig 2A) and light cycle (Fig 2B) for 14, 15, 18 and 19 wks of age (S1 and S2 Files). We also analyzed the influence of the activity stage. Whereas our previous study revealed alterations in hippocampal gamma frequency bands, no significant alterations in the motor cortex lower gamma range of 30–50 Hz range could be detected. For the 50–70 Hz range (Fig 2C and 2D), a statistical trend was observed in active females in the light cycle at the age of 18 wks (4.134 ± 0.399 v. 5.688 ± 0.655, p = 0.0715, Fig 2D_III_).

### Beta frequency analysis in controls and APPswePS1dE9 mice

**Fig 3:**
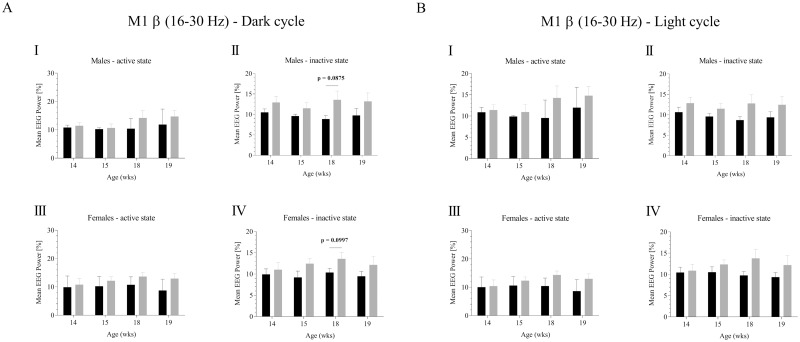
Beta frequency analysis of cortical M1 EEG recordings in controls and APPswePS1dE9 mice. The mean relative beta EEG power [%] was calculated FFT based for males and females considering potential circadian rhythmicity (dark phase (**A**), light phase (**B**)). Frequency analysis was performed for all four ages (14, 15, 18, 19 wks). Black, controls; gray, APPswePS1dE9. Note that only animals displaying highest quality EEGs (no EMG/ECG contamination) were finally included into the analysis. For sample size see original data.

Finally, we analyzed beta (16–30 Hz) frequency power in APPswePS1dE9 (Fig 3, S1 and S2 Files). As for theta and both gamma frequency bands, no significant changes could be detected for the beta range. Statistical trends were observed for inactive males and inactive females in the dark cycle at 18 wks of age (8.870 ± 0.869 v. 13.583 ± 2.140, p = 0.0875, Fig 3A_II_ and 10.356 ± 0.966 v. 13.583 ± 1.472, p = 0.0997, Fig 3A_IV_).

## Discussion

Alzheimer’s disease is a neurodegenerative disorder that is accompanied by neural cell loss that ultimately results in neural network dysfunction, such as dysrhythmia and / or aberrant excitability. We have previously reported that male APPswePS1dE9 exhibit a reduction in hippocampal theta at an age of 14 wks that vanished while animals are getting older [31]. In contrast, females exhibited a hippocampal theta power reduction at later stage of 18 and 19 wks of age. For both genders gamma activity displayed antithetic behavior. Importantly, an activity dependent analysis further illustrated that the antithetic theta-gamma power distribution in females was most prominent in the inactive state [31]. These results suggest that hippocampal theta alterations are most likely to be related to type II theta as the latter is associated i.a. with alert immobility [22, 27, 31, 37–40]. Disruption of theta activity results in spatial memory deficits, whereas the restoration of theta-like rhythmicity restores learning capabilities in rats [41]. We conclude that inactive mice in both the dark and light phase predominately exhibit alert-immobility which is accompanied by atropine-sensitive type II theta [42, 43]. Thus, our data suggest that female APPswePS1dE9 mice in particular demonstrate reduced atropine-sensitive type II theta at later stages which is likely to be due to septohippocampal impairment during AD development. In summary, hippocampal theta and gamma architecture in APPswePS1dE9 mice turned out to be gender, age and activity dependent. Most importantly, these findings and other studies [21, 30] clearly demonstrate that EEG alterations in APPswePS1dE9 mice already occur at the age of 2.5–4 months when plaques formations initiates, i.e. long before clinical signs of dementia such as cognitive decline become obvious.

Given this critical time window in early EEG alterations, we investigated potential similar alteration in motor cortical theta, beta and gamma frequency activity in the APPswePS1dE9 mouse model of AD in this study. Sensorimotor cortex excitability can be altered in Alzheimer patients [44, 45] and mouse models such as APPswePS1dE9 also exhibit motor impairments at late AD stage at 12 months of age [46]. The central question we addressed here is whether early cortical power alterations described in the frontal cortex of 16–17 wks old APPswePS1dE9 mice [30] are also present in cortical M1 deflections.

Notably, our study was carried out under physiological, i.e. spontaneous, conscious and un-restrained long-term recording conditions taking into account again gender, age and the activity status of the experimental animals. Although we could observe statistical trends in theta, beta and gamma power (Figs 1–3), no significant alterations were detected for any experimental constellation. Several aspects are likely to be responsible for this phenomenon. Pathohistologically, the Aβ burden of aged (up to 12 months) APPswePS1dE9 females appears to be more severe than in old (up to 12 months) males [24, 47–49]. Investigation of Aβ dynamics in this AD mouse line revealed further age- and gender-related specificities. In 4 months old APPswePS1dE9 mice, brain Aβ load has been reported to be dominated by Aβ_1–40_ rather than Aβ_1–42_ [24, 47]. Upon 6 months of age, Aβ ratio shifts towards Aβ_1–42_ that is maintained until end of life. In addition, a recent study demonstrated that senile plaques in the cortex and hippocampus can be detected in 3 months old males but barely in females of the same age which means that Aβ plaques occur earlier in males [50].

Gurevicius et al. [30] performed initial power analysis in the frontal cortex in female APPswePS1dE9 mice of 16–17 wks of age. Their non-telemetric short-term daytime recording of 1h revealed an increase in alpha, beta and gamma power. It is widely accepted that the frontal cortex and temporal lobe are early affected in AD, whereas the primary motor, sensory, and visual isocortical areas are lately involved which corresponds to the sparing of motor, sensory, and primary visual functions [51–54]. This is also reflected by the three stage (A-C) system of Braak and Braak [53]. In general, the involvement of multimodal high-order association isocortical areas is responsible for the progressive impairment of cognitive capabilities, including executive dysfunction (prefrontal cortex), apraxias (parietal cortex), visuospatial navigation deficits (occipitoparietal cortex), visuoperceptive deficits (occipitotemporal cortex), and semantic memory.

[30] hypothesized that multiple factors including basal forebrain projections and local hyperexcitability of cortical pyramidal cells based on increased resting membrane potential [21] may account for the observed alteration in frontal cortex gamma power. Studies by Garcia-Marin et al. [55] in the cortex (auditory cortex, visceral cortex, somatosensory cortex and visual cortex) of 12 months old male APPswePS1dE9 mice suggested that there is a lack of GABAergic perisomatic synapses of basket cells on the surfaces of cortical pyramidal neurons that are in close contact with amyloid plaques. As perisomatic GABAergic synapses exhibit a dominant influence on the output behavior of pyramidal neurons, their structural impairment may result in hyperactivity of the neurons in close proximity to amyloid plaques. Gamma-band rhythmogenesis is known to be inextricably tied to perisomatic inhibition, particularly via GABA A receptors [56]. However, as interneurons primarily determine the power of faster oscillations such as gamma [57–59], it seems unlikely that interneuronal pathology is solely responsible for the observed cortical gamma increase reported by [30] and [60].

## Conclusion

Our gender, age and activity dependent analysis of motor cortex theta, beta and gamma power in young 14–19 wks old APPswePS1dE9 mice did not reveal any alterations compared to the age-matched severe changes in the prefrontal cortex of this model reported previously [30]. Given the temporal pathohistological architecture in AD described above it seems likely that early sparing of motor cortex areas in amyloid plaque formation accounts for the preservation of theta, beta and gamma activity in the motor cortex of APPswePS1dE9 mice of 14–19 wks of age.

In summary, these findings further underline that characterization of AD specific EEG fingerprints or EEG biomarkers requires a sophisticated analysis of gender, age, activity and selected brain areas as spatial, e.g. cortical differences in frequency alterations can be tremendous.

## Supporting Information

S1 Fig FFT based EEG analysis. Representative 10 sec EEG segments are displayed FFT based up to 70 Hz. Representative amplitude spectra [V] are displayed for male APPswePS1dE9 and control mice at the age of 14 wks for the dark cycle for both the active and inactive state.
(PDF)

S1 File Relative cortical power of different EEG frequency bands during the dark cycle. Relative power values for both males and females are displayed using GraphPad Prism. Recordings which exhibited EMG artefacts (due to motor activity) or electrical artefacts and did not meet strict EEG quality criteria were not incorporated in the analysis (see Materials and Methods section).
(PZFX)

S2 File Relative cortical power of different EEG frequency bands during the light cycle. Relative power values for both males and females are displayed using GraphPad Prism. Animals which exhibited EMG artefacts (due to motor activity) or electrical artefacts and did not meet strict EEG quality criteria were not incorporated in the analysis (see Materials and Methods section).
(PZFX)

## Abbreviations

Aβ: amyloid beta
AD: Alzheimer’s disease
APP: amyloid precursor protein
BACE: beta-site amyloid precursor protein cleaving enzyme 1
CA: cornu ammonis
CNS: central nervous system
EEG: electroencephalogram
ECoG: electrocorticogram
FFT: Fast-Fourier-Transformation
i.p.: intraperitoneal
PS: presenilin
