# Supplementary figures and images for: Motor Cortex Theta and Gamma Architecture in Young Adult APPswePS1dE9 Alzheimer Mice
Source: PLoS One. 2017 Jan 10;12(1):e0169654. doi: 10.1371/journal.pone.0169654 (PMC5224826; doi:10.1371/journal.pone.0169654)

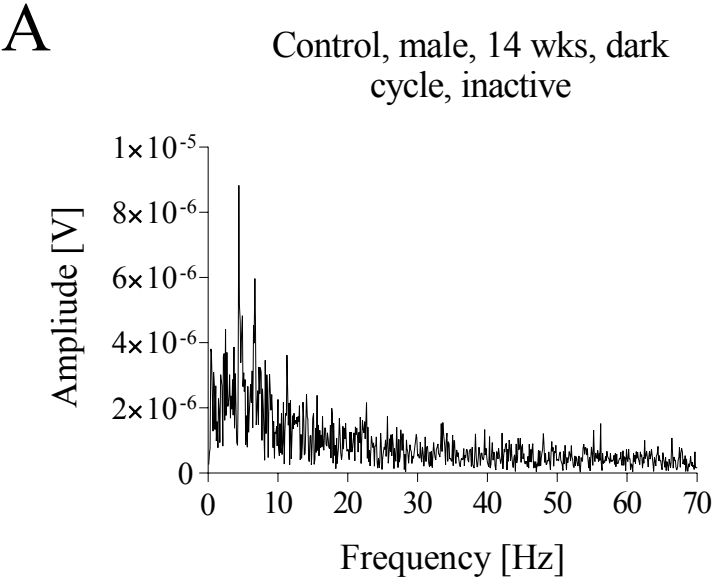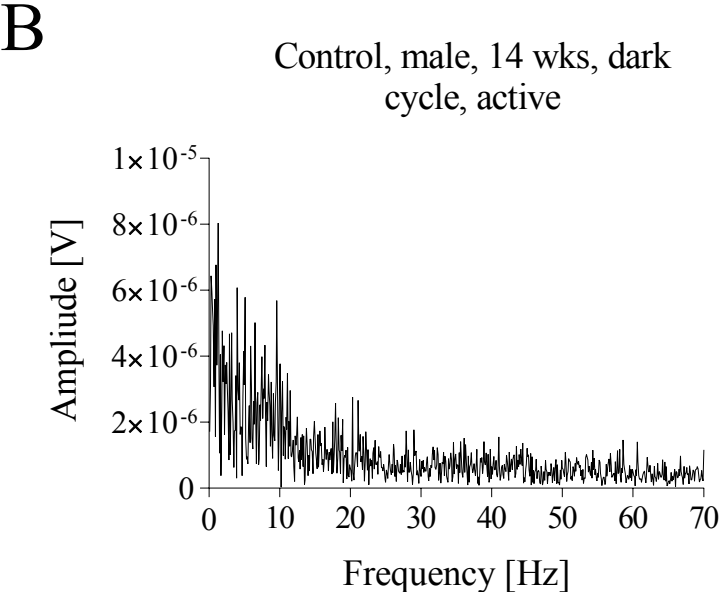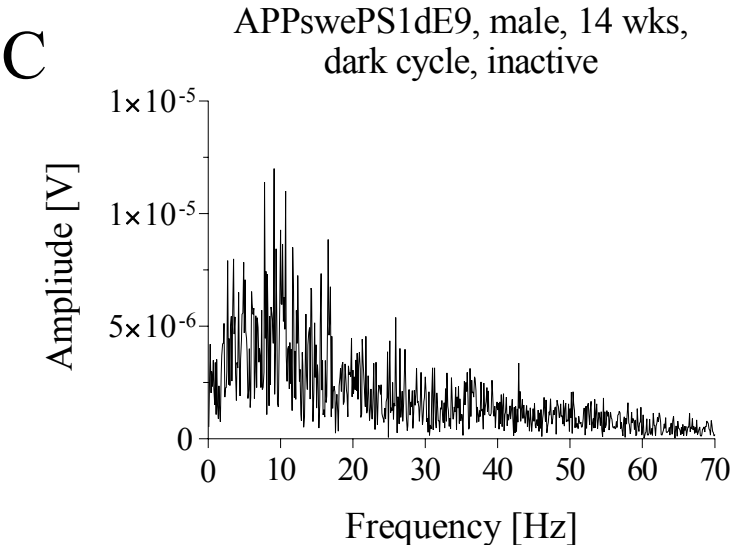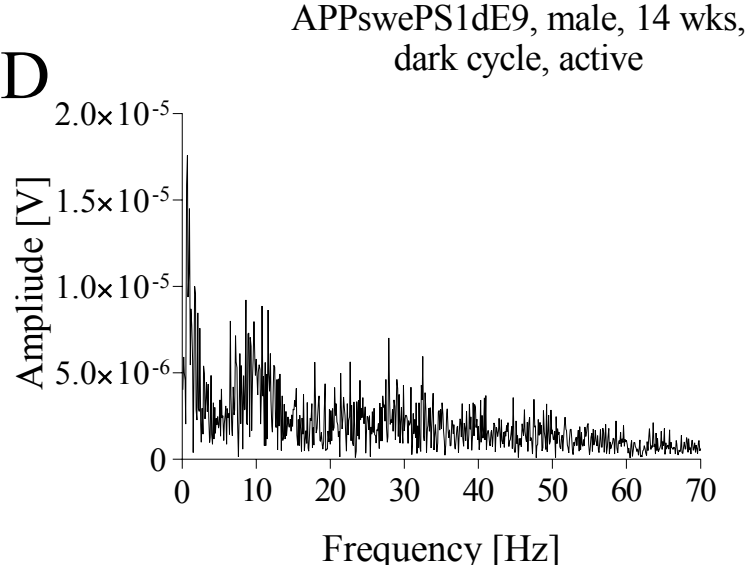

Supplement: S1 Fig — Representative 10 sec EEG segments are displayed FFT based up to 70 Hz. Representative amplitude spectra [V] are displayed for male APPswePS1dE9 and control mice at the age of 14 wks for the dark cycle for both the active and inactive state. (PDF) [file pone.0169654.s001.pdf]
